# Home quarantine during COVID-19 blunted childhood trauma-related psychiatric symptoms in Chinese college students

**DOI:** 10.3389/fpubh.2023.1073141

**Published:** 2023-05-25

**Authors:** Wenjuan Hong, Qiang Wang, Qinghong Hou, Nan Zhao, Ruoling Wang, Yan Bai, Chengping Hu, Weiqing Liu

**Affiliations:** ^1^Clinical Research Center for Mental Disorders, Shanghai Pudong New Area Mental Health Center, Tongji University School of Medicine, Shanghai, China; ^2^Department of Student Mental Health, Dianchi College, Yunnan University, Kunming, China; ^3^Department of Psychiatry, First Affiliated Hospital of Kunming Medical University, Kunming, China

**Keywords:** home quarantine, mental health, childhood trauma, college student, social supports

## Abstract

**Background:**

Childhood trauma confers risks to mental health. However, little is known about whether home quarantine (HQ) during the coronavirus disease 2019 (COVID-19) pandemic exaggerated or mitigated the effect of childhood trauma on mental health.

**Objective:**

To examine the modulating effects of prior childhood traumas on the longitudinal changes of psychiatric symptoms in college students before and after HQ during the pandemic.

**Methods:**

This was a two-wave longitudinal study on the mental health of 2,887 college students before and after HQ during the COVID-19 pandemic. The relationships between the changes in the Patient Health Questionnaire-9 (PHQ-9), Symptom Checklist-90 (SCL-90), 16-item Prodromal Questionnaire (PQ-16), Childhood Trauma Questionnaire (CTQ), and Social Support Rating Scale (SSRS) scores were analyzed.

**Results:**

The students with childhood trauma showed a significantly greater decrement in psychiatric symptoms after HQ (*F* = 17.21, 14.11, 18.87, and 17.42 for PHQ-9, PQ-16 objective and distress, and SCL-90, respectively). The correlation coefficients between the CTQ and these symptoms scales were significant at baseline (*r* = 0.42, 0.34, 0.37, and 0.39), and decreased after HQ (*r* = 0.17, 0.20, 0.18, and 0.19). The decrement of depressive, psychotic, and overall symptoms was positively correlated with the scores of the CTQ (*r* = 0.08–0.27) but negatively correlated with SSRS (*r* = −0.08−−0.14). Multilinear regression analysis confirmed the results of the CTQ and SSRS regarding the modulation of the dynamic changes in psychiatric symptoms. A constructed structural equation model indicated that the total effects of childhood trauma on decreased psychiatric symptoms were partly mediated by lower baseline social support.

**Conclusion:**

Home quarantine during the COVID-19 pandemic could blunt the adverse effects of childhood trauma on mental health, especially for prodromal psychotic symptoms in college students. Changes in relative deprivation and social support may be mediating factors.

## Introduction

The coronavirus disease 2019 (COVID-19) pandemic has lasted for more than 2 years and is still affecting the whole world. To reduce its transmission, the first response for most countries was to enter full lockdown. These lockdowns included travel bans, mandatory home quarantines (HQs), and temporary closures of nonessential businesses. Currently, the most common practices to prevent COVID-19 transmission are maintaining social distance, wearing face masks, reducing social activities, screening for virus carriers within the population, and committing to HQ and self-isolation (1). Consequently, the pandemic has profoundly changed the normality of social and family lives and brought significant mental health challenges to many populations (2–4). These mental health challenges are especially notable for college students who are in a critical developmental period, transitioning from late adolescence to emerging adulthood, entering a stage of decreased attachment, and becoming increasingly antagonistic with their parents (5–10). Therefore, it is worthwhile to explore the mental health dynamics of college students and their related modulating factors during HQ with the students’ families in the pandemic.

It is well known that young college students comprise a population that is particularly vulnerable to stress and mental health concerns (8). Two worldwide surveys separately found that 20.3 and 35% of the respondent college students had experienced at least one mental health disorder before the pandemic (8, 11), among which phobia (9.0%) and major depressive episode (21.2%) account for the most prevalent disorders, respectively. But both surveys omitted the psychotic disorders or prodromal psychotic symptoms, which reaches onset peak during late adolescence and generally have a long-lasting substantial impact on the individuals (12). However, the rates of self-reported “prodromal syndrome” in the college population can be as high as 25% (13). This high prevalence of mental health concerns among college students may be worsened by such stressors related to the COVID-19 pandemic as fear of being infected, intolerance of uncertainty, burnout, constraints on social activities, and radical lifestyle changes (2, 14, 15).

During the COVID-19 pandemic, dramatic changes in living environments and surrounding interpersonal relationships stemming from HQ may constitute a major stressor. Therefore, some related factors, such as prior stressful experiences in family settings and the transition of social support from outside to inside the family, can modulate the stress responses and mental health status during HQ. Indeed, a recent study found that family cohesion can effectively alleviate the stress consequences of the COVID-19 pandemic (16). In contrast, adverse childhood experiences (childhood traumas) are related to stronger PTSD symptoms (17), severer depressive symptoms (18), higher levels of destabilized autonomic reactivity, and mental health problems (19). In addition, several studies have suggested that a collapsed social supporting system, feelings of loneliness, damaged family function, and past traumatic experiences in the family environment may be important predictors of more severe psychiatric symptoms during HQ in the pandemic, indicating the importance of a social support system in coping with stress and maintaining mental wellbeing (18, 20–24).

Childhood trauma and social support can profoundly modulate the stress response derived from HQ and its related psychiatric symptoms in college students. During HQ, people generally are required to stay at home with their family members for several weeks or months. Obviously, in a long-term closed environment, support from the wider family and social connections are restricted; therefore, the intra-family relationships and related support are the most accessible option and become more important than ever in how it affects the capacity to cope with stress during HQ (17, 23). This is especially critical for young college students who have experienced abuse or neglect in childhood and developed a decreased attachment and alienated relationship with their parents and peers as they grow older. These college students might be more sensitive to the radical changes in their lives and social connections during HQ because they generally have fewer resources for seeking support (25, 26). On the other hand, during HQ, the core family members mostly live together and spend more time communicating with each other and confronting challenges together, and can even closely observe each other’s social or work roles normally conducted outside of the family (27). Therefore, HQ can objectively provide a rare opportunity for the core family members to communicate, understand, and support each other in a commonly challenging pandemic environment, and hence may fix the strained or alienated relationships stemming from past physical or emotional neglect or parental abuse (28). Thus, it is hypothesized that prior childhood trauma experiences and social support could modulate the stress sensitivities of college students to HQ, and the social support level may partly mediate the impact of childhood trauma.

Childhood trauma has long been reported to be associated with many types of mental health problems during adulthood (29, 30), and it has recently been shown to predict elevated levels of multiple psychiatric symptoms during the COVID-19 pandemic. However, most of the current studies were based on cross-sectional data, which could find associations between childhood trauma and psychiatric symptoms during the COVID-19 pandemic (17, 19). However, these studies could not separate the already established relationship between childhood trauma and psychiatric symptoms before COVID-19, and they focused on the modulating effects of childhood trauma on the longitudinal changes of psychiatric symptoms derived from HQ, either positive or negative. Consequently, a longitudinal study that incorporates both baseline and post-pandemic mental health status may help decipher the role of childhood trauma in the mental health dynamics of college students during HQ.

Other factors may also modulate the stress response and mental health dynamics during HQ (or other isolations) in the COVID-19 pandemic. A study on the psychosocial effects of the COVID-19 pandemic in adults identified the risks and protective factors that predict changes in mental health status, and it found that those who were women, younger, students, unemployed, or had a prior psychiatric history were at risk of increased anxiety and depression symptoms (31). Another study on the psychosocial predictors of mental health in Italy during national quarantine against the COVID-19 pandemic, showed that lower coping efficacy, more worrying, and negative attitudes toward quarantine measures were predictors of mental health problems (32). Moreover, a study among young people in six different countries revealed that being female, being in contact with a person with mental illness, being quarantined, and internet usage are significant predictors of stress, anxiety, and depression (33). However, most of the studies on mental health concerns and their modulating factors during HQ or COVID-19 were cross-sectional or qualitatively designed and could not specifically focus on the net changes of psychiatric symptoms before and after HQ to exclude the prior established correlations between psychiatric symptoms and their modulating factors (4). As a result, a longitudinal study is needed to address the effects of adverse childhood experiences, social support, and demographic characteristics on college students’ mental health dynamics before and after HQ in the COVID-19 pandemic.

This present study conducts a two-wave longitudinal study on the effects of adverse childhood experiences and social support on the dynamic changes of psychiatric symptoms before and after HQ, using a relatively large sample of Chinese college students.

## Methods

### Study design and population

Data were collected before (from 8 a.m., September 17, 2018, to 9 p.m., September 27, 2018) and after the COVID-19 outbreak (from 5 p.m., May 10, 2020, to 9 p.m., May 23, 2020). The participants were initially recruited during the school-managed freshman college student mental health survey program Uncapitalized from 10 colleges in Yunnan University of China during September 2018. All first-year students were required to finish a Symptom Checklist-90 (SCL-90) in the program (34). Using a convenient sampling method, a total of 2,887 students were recruited in this study and finished additional self-reported questionnaires, including demographic, Patient Health Questionnaire-9 (PHQ-9), 16-item Prodromal Questionnaire (PQ-16), Childhood Trauma Questionnaire (CTQ), and Social Support Rating Scale (SSRS) *via* the specialized online Chinese questionnaire tool^1^ distributed through WeChat (a Chinese mainstream social media platform). After a quality control process, 349 participants were excluded due to the short time (under 240 s) spent finishing the online questionnaires. Among the remaining 2,538 participants, there were 2,485 of them that had student ID numbers that matched SCL-90 data in the university’s mental health intra-website system. The 2,538 participants were invited to take the re-test in May 2020, immediately after finishing their 3-month HQ (from February to May 2020) during the COVID-19 pandemic and returning to school. A total of 2,419 students took the re-test, during which 2,076 of them finished the PHQ-9 and PQ-16, and they self-reported the percentages of their grades during the last school year and place of residence during HQ, all *via* the same online questionnaire tool as before. Of the 2,419 students, a total of 2,330 finished the SCL-90 *via* the same intra-website system. All participants signed the online consent forms before completing the self-report questionnaires. The changes in psychiatric symptoms before and after HQ were calculated by baseline score minus the after-HQ score for each scale. All participants gave their informed consent for inclusion before participating in the study. The study was conducted following the Declaration of Helsinki, and the protocol was approved by the Institutional Review Board of the First Affiliated Hospital of Kunming Medical University.

### Measures

The PHQ-9 was adopted to assess participants’ symptoms of depression during the last 7 days before the questionnaire was taken. It used the DSM-IV diagnostic criteria for evaluating depressive symptomatology (e.g., sleep, concentration, energy problems, low self-esteem, and anhedonia) on a four-point scale ranging from 0 (not at all) to 3 (nearly every day). The PHQ-9 has been widely used in different populations to screen depressive patients (35, 36). The validity and reliability of the PHQ-9 has been tested to be acceptable in a Chinese general population (37). In addition to its utility as a short screener, the PHQ-9 also captures depression severity. Total scores of 0–4, 5–9, 10–14, 15–19, and 20–27 represented none (sub-threshold), mild, moderate, moderately severe, and severe depression, respectively (36). Individuals with PHQ-9 scores of 5 or above were classified as supra-threshold, and those with scores of 10 or above were classified as having obvious depressive symptoms.

The PQ-16 was adopted to assess the presence of positive and negative prodromal psychotic symptoms. Nine items assessed perceptual abnormalities and hallucinations; five assessed unusual thought content, delusional ideas, and paranoia; and two assessed negative symptoms. Items were first answered on a two-point scale: objective score, 1 = true, 0 = false. Then, if the item was true, the extent and severity of distress were measured: 0 = None, 1 = Mild, 2 = Moderate, and 3 = Severe (38). The total objective and distress scores of the PQ-16 were calculated. The cutoffs for PQ-16 objective and distress scores were set as 7 and 8, respectively, to define the individuals with or without definite prodromal psychotic symptoms, according to a published validity and reliability study of a Chinese sample (39).

The SCL-90 is a 90-item questionnaire that assessed a broad range of psychological problems and symptoms of psychopathology in the last week before taking the questionnaire (40). Each item was scored on a scale from 0 to 4 based on how bothered students were by each item, with 0 = not at all, 1 = a little bit, 2 = moderately, 3 = quite a bit, and 4 = extremely. The SCL-90 included 10 primary factors (somatization, obsessive–compulsive, interpersonal sensitivity, depression, anxiety, hostility, phobic anxiety, paranoid ideation, psychoticism, and additional items) (41). The Chinese version of the SCL-90 used in this study has acceptable validity and satisfactory reliability (42). The participants were divided into three identical groups according to their baseline total SCL-90 score (lower third, medium third, and top third).

The CTQ is a 28-item self-report inventory that rates the severity of five factors of childhood trauma [physical abuse (PA), physical neglect (PN), emotional abuse (EA), emotional neglect (EN), and sexual abuse (SA)] to screen for a positive history of abuse or neglect in childhood (43). A five-point Likert scale was used for each item. A high total score represented a highly traumatic childhood. If any one of the five factors reached their threshold (PA ≥ 10, PN ≥ 10, EA ≥ 13, EN ≥ 15, or SA ≥ 8), the individuals would be rated as CTQ+; otherwise, they would be rated as CTQ− (44). The Chinese version of the short-form CTQ has been widely used in different populations, and its validity and reliability have been confirmed (22, 45).

The SSRS contained 10 items that measure objective and subjective social support from the family, relatives, friends, peers, neighbors, community, and organizations, and it also measures the utilization of these supports (46, 47). Item scores of the SSRS were added, generating a total support score ranging from 12 to 66. Higher SSRS total scores indicated stronger social support, and a score of 0–22, 23–44, and 45–66 indicated low-level, medium-level, and high-level social support, respectively. The SSRS has been widely used in the Chinese population, and its validity and reliability have been confirmed in different samples (22, 48, 49).

### Statistical analysis

Percentages were analyzed for categorical variables and mean values for continuous variables. The difference in demographic characteristics and psychiatric symptom ratings of the study population between baseline and after HQ were analyzed with the Chi-squared test for categorical variables and the Wilcoxon matched-pairs signed-rank test for continuous variables. An independent samples *t*-test was used to compare the number of changes in questionnaire scores between students with and without childhood trauma. When controlling for demographic variables, Spearman’s partial correlations were computed to compare the associations between the different CTQ and SSRS factors and psychiatric symptoms. The *p* values in multivariable correlation analysis were Bonferroni corrected. Multilinear regression was used to analyze the contribution of demographic characteristics, CTQ, and SSRS to decreased psychiatric symptoms. A structural equation model (SEM) was built based on the hypothesis of the relationships between childhood trauma, social support, and the longitudinal changes in mental health before and after HQ, using IBM SPSS AMOS 24.0 software. The direct and indirect effects of childhood trauma and social support on the decreased psychiatric symptoms were analyzed using maximum-likelihood covariance estimation. All other statistical analyses were performed using IBM SPSS Statistics V23.0 software. A value of *p* < 0.05 was regarded as being statistically significant.

## Results

### Demographic characteristics of the participants

A total of 2,538 valid participants were followed up in the study population to observe the dynamic changes in their psychiatric symptoms under stressful events (COVID-19 in this study) and to explore the correlated predicting factors. Most participants (95.27%) joined the re-test 20 months after the baseline test (immediately after HQ), as shown in Table 1. Most participants were women at baseline and after HQ (70.76 and 72.13%, respectively). The average age of the participants was 18.83 at baseline, representing a typical first-year college student’s age. The place of residence during the HQ period was self-reported as city, town, or village in 12.3, 38.7, and 49.0% of assessments, respectively. The 3-month HQ changed the studying model of the students from offline to online, which may have brought different challenges for different students. Therefore, it was evaluated if the participants’ academic performance would have impacted the dynamics of psychiatric symptoms during the period. The self-reported percentage of grades during the past school year was collected after the HQ. Results showed that 36.56% of the participants were self-reported to be in the top third scoring grades, while 17.3% of them were self-reported to be in the lower third.

**Table 1:** Demographic characteristics and psychiatric symptom ratings of the cohort at baseline and after HQ.

Variable	Baseline		After HQ		*χ^2^/Z*s	*p* value
	*N*	%/(mean ± SD)	*N*	%/(mean ± SD)		
Participants	2,538		2,419	95.27%		
Sex	2,538		2,419		4.41	0.11
Male	742	29.24%	674	27.87%		
Female	1,796	70.76%	1,744	72.13%		
Age	2,538	18.83 ± 0.95				
Place of residence during HQ			2,076			
City			272	12.33%		
Town			799	38.68%		
Village			1,347	48.99%		
Percentage of grades			2,076			
1–33%			759	36.56%		
34–66%			951	45.818%		
67–100%			366	17.3%		
CTQ	2,538	38.64 ± 8.74				
CTQ (+)	588	76.83%				
CTQ (−)	1,950	23.17%				
SSRS	2,538	39.29 ± 5.42				
Low-level	6	0.24%				
Medium-level	2,109	83.10%				
High-level	423	16.67%				
PHQ-9	2,076^*^	2.50 ± 2.80	2,076	1.88 ± 2.82	*−9.84*	*<0.001*
					*17.20*	*<0.001*
0–4	1,675	80.68%	1,773	85.40%		
5–9	347	16.71%	255	12.28%		
10-	54	2.60%	48	2.31%		
PQ-16_Ojectvie	2,076^*^	2.62 ± 2.62	2,076	1.28 ± 2.04	*−22.17*	*<0.001*
					*55.57*	*<0.001*
0–6	1,886	90.85%	2,003	96.48%		
7-	190	9.15%	73	3.52%		
PQ-16_Distress	2,076^*^	1.24 ± 2.38	2,076	0.64 ± 1.80	*−12.69*	*<0.001*
					*7.68*	*0.006*
0–7	2,018	97.21%	2,044	98.46%		
8-	58	2.79%	32	1.54%		
SCL-90	2,283^*^	132.24 ± 31.28	2,283	118.52 ± 29.63	*−21.01*	*<0.001*
					*282.12*	*<0.001*
90–114	761	33.33%	1,316	57.64%		
115–139	764	33.46%	550	24.10%		
140-	758	32.20%	417	18.27%		

HQ, home quarantine; CTQ+, childhood trauma questionnaire; SSRS, social support rating scale; PHQ-9, patient health questionnaire-9; PQ-16, 16 items prodromal questionnaire; SCL-90, symptom checklist-90; and N/A, not available.

* Only the participants who responded in the re-test (N for After HQ) for corresponding scales were included in the Pre-Post comparisons and the subsequent statistical analysis.

### Psychiatric symptoms generally decreased after home quarantine

As shown in Table 1, depressive symptoms were quite common in the participants at baseline, with 19.23% of the PHQ-9 total scores higher than 5, the lower threshold for mild depressive symptoms. Surprisingly, this proportion of depressive individuals decreased to 14.6% after the 3-month HQ period (*χ^2^* = 17.66, *p* < 0.001). The average PHQ-9 score also significantly decreased after HQ in comparison with that at baseline (*Z* = −9.84, *p* < 0.001). A more significant decrease was found for prodromal psychotic symptoms, as indicated by the means of both the objective and distress factors of PQ-16, decreasing by nearly 50% after the HQ (*Z* = −22.17 and − 12.69, respectively, both *p* < 0.001). The percentages of participants with supra-threshold prodromal psychotic symptoms were 9.14 and 2.98% for objective and distress factors, which decreased to 3.52 and 1.54%, respectively, after HQ (*χ^2^* = 58.52, *p* < 0.001 and *χ^2^* = 9.15, *p* = 0.002, respectively). The same trends were observed for overall psychiatric symptoms, represented by a significant decrease in the average SCL-90 total scores, from 132.42 to 118.62 (*Z* = -21.01, *p* < 0.001). Accordingly, 57.2% of the participants had an after-HQ SCL-90 score that was less than the lower third based on the baseline scores, while only 17.73% of the participants had an SCL-90 score higher than the upper third after HQ, which indicated a downward shift in the overall severity of psychiatric symptoms after HQ (*χ^2^* = 291.39, *p* < 0.001).

### Participants with childhood trauma experiences benefit more from home quarantine with decreasing psychiatric symptoms

**Figure 1:**
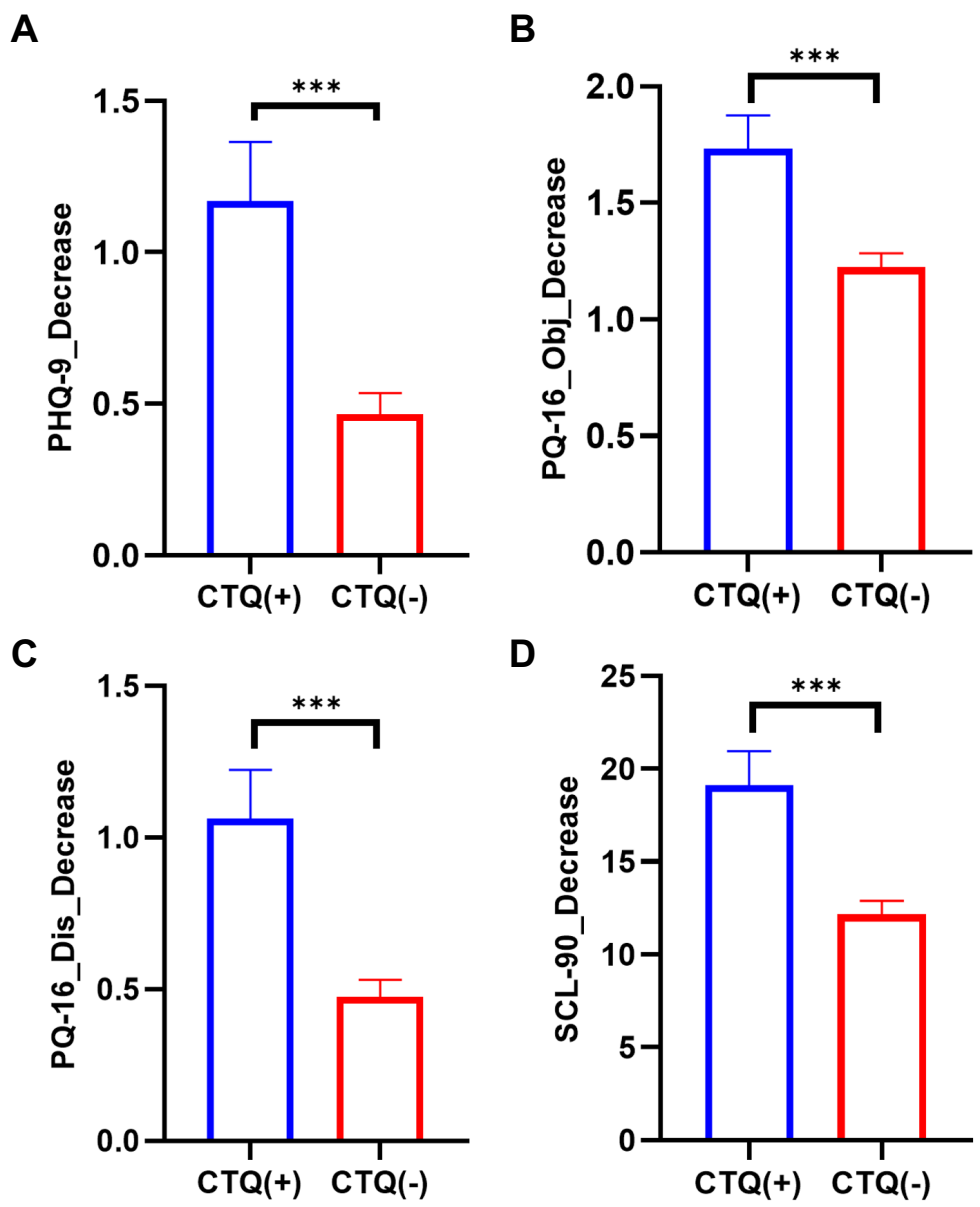
Participants with childhood trauma experiences benefit more from HQ in decreasing psychiatric symptoms. Comparison of the decreased PHQ-9 **(A)**, PQ-16 objective **(B)**, PQ-16 distress **(C)**, and SCL-90 **(D)** scores between the participants with childhood trauma experiences (CTQ+, *n* = 461 for PHQ-9 and PQ-16, *n* = 515 for SCL-90) and the participants without that (CTQ−, *n* = 1,615 for PHQ-9 and PQ-16, *n* = 1,769 for SCL-90). HQ, home quarantine; PHQ-9, patient health questionnaire-9; PQ-16, 16 item prodromal questionnaire; SCL-90, Symptom Checklist-90; and CTQ, childhood trauma questionnaire; ^***^p < 0.001.

To further explore if past adverse experiences from the childhood family environment would impact the dynamic changes in psychiatric symptoms in that very same environment (HQ in this study) during COVID-19, the differences in the decreased PHQ-9, PQ-16, and SCL-90 scores between the participants (categorized as CTQ+ and CTQ−) were directly compared. Interestingly, the results (Figure 1) showed that CTQ+ participants had significantly larger decreased scores for all the PHQ-9, PQ-16 objective, PQ-16 distress, and SCL-90 questionnaires (*F* = 17.21, 14.11, 18.87, and 17.42, respectively, all *ps* < 0.001).

### Correlation between childhood trauma questionnaire, social support rating scale, and the decreased psychiatric symptoms

**Figure 2:**
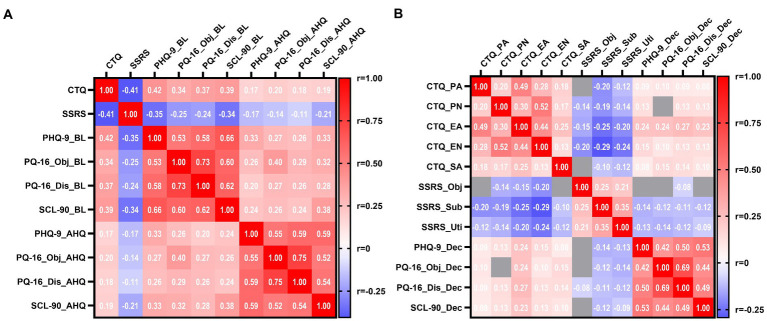
Correlations between CTQ, SSRS, and different psychiatric symptom scores. **(A)** Heat-map for Spearman’s *r* of correlation matrix between CTQ, SSRS total score, and different psychiatric symptom scores at baseline and after HQ. **(B)** Heat-map for Spearman’s r of correlation matrix between CTQ, SSRS factor scores, and decreased psychiatric symptoms scores (after HQ-baseline). All partial correlations were controlled for potentially confounding variables (age, sex, place of residence, and percentage of grades), and positive correlations were shown in red squares, and negative correlations were shown in blue on the heat-map. Most correlations were statistically significant even after Bonferroni correction (*p* < 0.05) except for several correlations indicated by gray squares in the heat-map. *n* = 1,943. CTQ, childhood trauma questionnaire; SSRS, social support rating scale; PHQ-9, patient health questionnaire-9; PQ-16_Obj, 16-item prodromal questionnaire objective factor; PQ-16_Dis, 16 items prodromal questionnaire distress factor; SCL-90, symptom checklist-90; BL, baseline; AHQ, after home quarantine; PA, physical abuse; PN, physical neglect; EA, emotional abuse; EN, emotional neglect; SA, sexual abuse; Obj, objective supports; Sub, subjective supports; Uti, utility of supports; Dec, decrease; and r, spearman’s correlation coefficient. ^#^*p* = 0.001 in CTQ-SSRS-PHQ-9 model; ^*^p = 0.002 in CTQ-SSRS-PQ-16; and CTQ-SSRS-SCL-90 models.

The partial correlation analysis between the CTQ, SSRS, and different psychiatric symptom scores was calculated. Significant correlations between CTQ, SSRS total score, and both baseline and after-HQ psychiatric symptom scores were confirmed (all *p* < 0.001), with nearly 2-fold higher correlation coefficients found for baseline than for after HQ in the CTQ and SSRS (Figure 2A), which indicated a significant decrease in the correlations after HQ. The correlations between the CTQ, SSRS factor scores, and the dynamic changes of the psychiatric symptoms before and after HQ (after HQ - baseline) were further examined. As shown in Figure 2B, all the five CTQ factors, three SSRS factors, and four psychiatric symptom dimensions were inter-correlated within each category (all *p* < 0.001). A weaker but significant positive correlation (all *p* < 0.05, after Bonferroni correction) between almost all the five CTQ factors and the PHQ-9, PQ-16 objective and distress, and SCL-90 score were observed even after controlling for potentially confounding variables (age, sex, place of residence, and percentage of grades). Among the five CTQ factors (PA, PN, EA, EN, and SA), EA was most strongly correlated with all the psychiatric symptoms. Two of the three SSRS factors (subjective supports and the utility of supports) were negatively correlated with all the CTQ factors and psychiatric symptoms, while objective supports were more weakly correlated with part of the CTQ factors (PN, EA, and EN) and PQ-16 distress scores.

### Multilinear regression of demographic characteristics between childhood trauma questionnaire and the social support rating scale on the decreased scores of psychiatric symptoms

Demographic characteristics, such as sex, age, the social environment of residence during the COVID-19 pandemic, and academic achievements of the college students may also have affected the sensitivity to stress and coping resources and may have impacted the effects of HQ on mental health. To explore the effects of those covariates on the dynamics of psychiatric symptoms, multilinear regressions of those demographic characteristics, CTQ, and SSRS on the decreased scores of psychiatric symptoms were executed. The regression models for all four decreased symptom scores (PHQ-9, PQ-16 objective and distress, and SCL-90) were significant (all *p <* 0.001). Among the included demographic characteristics, only sex and age were predictors for decreased PHQ-9 scores after HQ, with men predicting a less decreased PHQ-9 score (*B* = −0.359, *p <* 0.05) and higher ages predicting a much-decreased PHQ-9 score (*B* = 2.384, *p <* 0.05). In accordance with the results from the comparison between the CTQ+ and CTQ− subjects, multilinear regression results (Table 2) showed that higher CTQ scores could predict much-decreased PHQ-9 (*B* = 0.070, *p <* 0.001), PQ-16 objective (*B* = 0.047, *p <* 0.001), PQ-16 distress (*B* = 0.061, *p <* 0.001), and SCL-90 (*B* = 0.718, *p <* 0.001) scores, while higher SSRS scores were associated with less decreased psychiatric symptom scores (*B* = −0.053, *p <* 0.001; *B =* −0.045, *p <* 0.001; *B =* −0.031, *p <* 0.01; and *B =* −0.393, *p <* 0.05 for PHQ-9, PQ-16 objective, PQ-16 distress, and SCL-90, respectively).

**Table 2:** Multilinear regression of demographic characteristics, CTQ, and SSRS on the decreased scores of psychiatric symptoms.

Variable	PHQ-9		PQ-16_Objective		PQ-16_Distress		SCL-90	
	*B(SE)/R^2^*	*t/F*	*B(SE)/R^2^*	*t/F*	*B(SE)/R^2^*	*t/F*	*B(SE)/R^2^*	*t/F*
Sex	−0.359 (0.162)	−2.223^*^	0.193 (0.130)	1.479	0.061 (0.129)	0.472	0.013 (1.738)	0.007
Age	0.180 (0.075)	2.384^*^	−0.005 (0.061)	−0.247	0.031 (0.060)	0.507	−0.461 (0.812)	−0.568
Percentage of grades	−0.004 (0.003)	−1.520	−0.001 (0.002)	−0.285	0.002 (0.002)	0.877	0.010 (0.032)	0.328
Place of residence	0.192 (0.103)	1.872	−0.001 (0.083)	−0.009	0.031 (0.082)	0.378	0.828 (1.103)	0.750
CTQ	0.070 (0.009)	7.607^***^	0.047 (0.007)	6.406^***^	0.061 (0.007)	8.358^***^	0.718 (0.098)	7.322^***^
SSRS	−0.053 (0.014)	−3.690^***^	−0.045 (0.012)	−3.831^***^	−0.031 (0.012)	−2.644^**^	−0.393 (0.156)	−2.517^*^
Model summary	0.059	21.652^***^	0.043	15.412^***^	0.054	19.590^***^	0.045	15.412^***^

CTQ, childhood trauma questionnaire; SSRS, social support rating scale; PHQ-9, patient health questionnaire-9; PQ-16, 16 item prodromal questionnaire; SCL-90, symptom checklist-90; B, regression coefficient; and SE, standard error.

^***^p < 0.001; ^**^p < 0.01; and ^*^p < 0.05.

### Social support partly mediated the effects of childhood trauma on decreased patient health questionnaire-9, 16-item prodromal questionnaire, and symptom Checklist-90 scores

**Figure 3:**
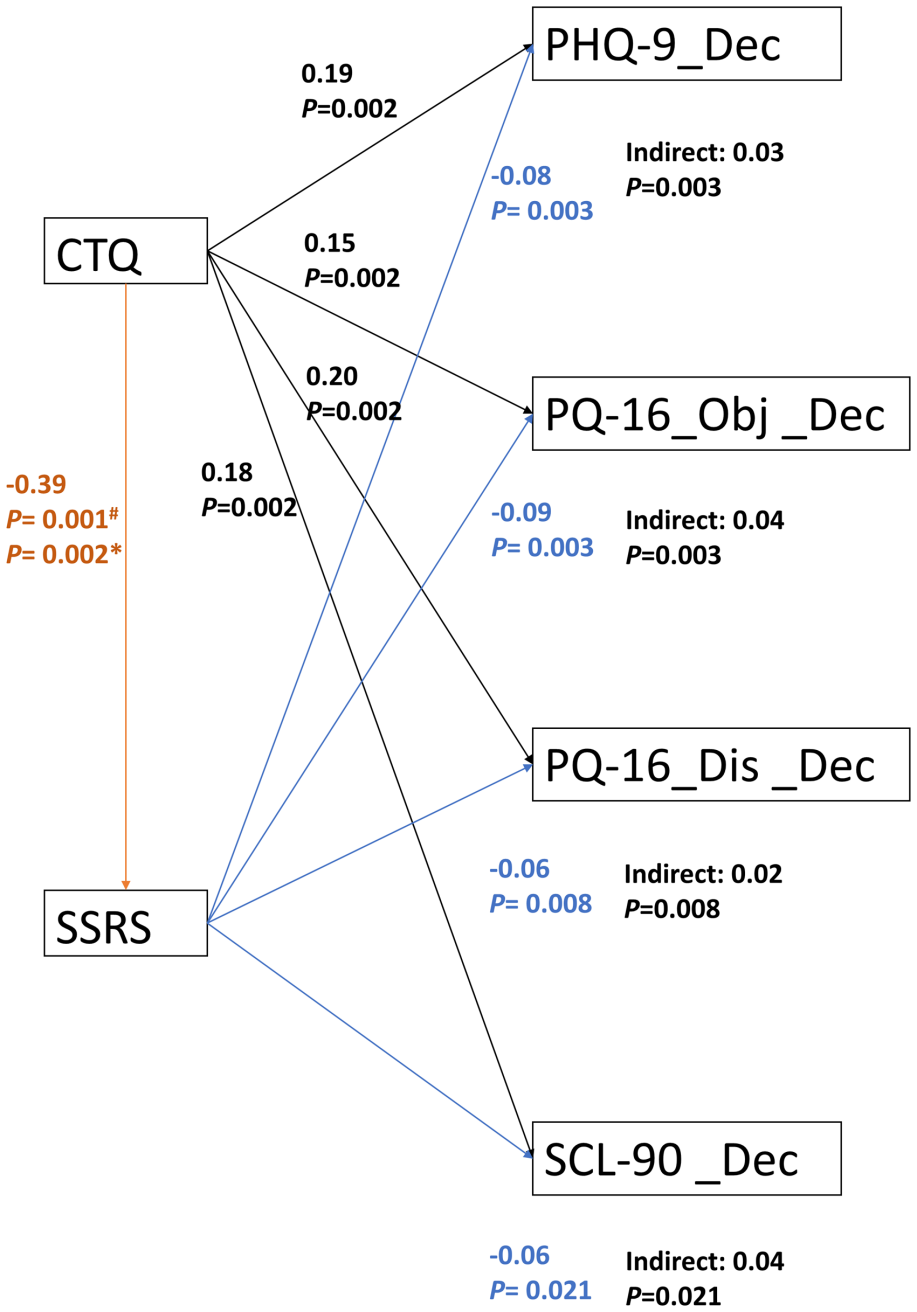
SSRS partly mediated the effects of CTQ on decreased psychiatric symptoms. The constructed SEM in SPSS AMOS indicated that the coefficient of each path was substantially significant. The standardized indirect path coefficients of SSRS on the decreased psychiatric symptoms were generally small but statistically significant. CTQ, childhood trauma questionnaire; SSRS, social support rating scale; PHQ-9, patient health questionnaire-9; PQ-16_Obj, 16-item prodromal questionnaire objective factor; PQ-16_Dis, 16-item prodromal questionnaire distress factor; SCL-90, symptom checklist-90; and Indirect, standardized indirect path coefficient. ^#^*p* = 0.001 in CTQ-SSRS-PHQ-9 model; ^*^p = 0.002 in CTQ-SSRS-PQ-16; and CTQ-SSRS-SCL-90 models.

Based on the association between the CTQ and SSRS scores and their correlation with the decreased psychiatric symptoms in multilinear regression analysis, a SEM was further constructed to examine the mediating effects shown by the SSRS between childhood trauma and the decreased psychiatric symptoms. As shown in Figure 3, the coefficients of each path were substantially significant. The standardized direct path coefficient of the CTQ on the SSRS was −0.39 (*p* = 0.001 in the PHQ-9 model, *p* = 0.002 in other models). The standardized direct path coefficients of the CTQ on the PHQ-9, PQ-16 objective, PQ-16 distress, and SCL-90 were 0.19 (*p* = 0.002), 0.15 (*p* = 0.002), 0.20 (*p* = 0.002), and 0.18 (*p* = 0.002), respectively. While the standardized direct path coefficients of the SSRS on the PHQ-9, PQ-16 objective, PQ-16 distress, and SCL-90 were − 0.08 (*p* = 0.003), −0.09 (*p* = 0.003), −0.06 (*p* = 0.008), and − 0.06 (*p* = 0.021), respectively. The standardized indirect path coefficients of SSRS on the decreased psychiatric symptoms were 0.03 (*p* = 0.003), 0.04 (*p* = 0.003), 0.02 (*p* = 0.008), and 0.04 (*p* = 0.021), respectively, indicating a small but significant mediating effect of the social support available between the childhood trauma and the decreased depressive and psychotic symptoms.

## Discussion

In summary, this study found that after HQ during the COVID-19 pandemic, the college students’ average PHQ-9 (depressive symptoms), PQ-16 objective and distress (prodromal psychotic symptoms), and SCL-90 total scores (overall psychiatric symptoms) decreased significantly compared with before the pandemic (Table 1). Correlation and regression analysis confirmed that more childhood trauma experiences and less baseline social support could predict a greater decrease in all psychiatric symptoms (Figures 1, 2B; Table 2). At the same time, being female or older correlated with relief from depressive symptoms (Table 2). Further analysis indicated that lower baseline social support partly mediated the effects of childhood trauma on the decreased psychiatric symptoms after HQ. These findings provide new insights into the mental health dynamic changes before and after the HQ of college students and the roles of childhood trauma and social support in modulating the mental health dynamics.

Particularly, this study found that depressive symptoms were relatively common before the pandemic, represented by 19.23% of the participants who scored as having mild depressive symptoms or above in the PHQ-9. Intriguingly, this prevalence of depressive symptoms goes down to 14.6% after the 3-month HQ period. This trend also holds for the overall mental health levels and prodromal psychotic symptoms, which reduced by nearly half in the PQ-16 objective and distress scores. The results of this study were partly supported by some studies with similar dynamic comparisons of mental health status before and after the pandemic. By making use of a subsample of a larger, ongoing first-year student emotional health and wellness study, Copeland et al. (6) reported that externalizing problems and problems with attention, but not internalizing symptoms, were increased in college students after the onset of COVID-19, while living with parents was a significant protective factor against anxiety. Similarly, Stinson et al. (50) drew participants from a larger pre-existing study population and found that the adolescents reported improved mood symptoms and COVID-19-related distress across the three waves of data collection after the COVID-19 pandemic, and the authors speculated that the improvement could be explained by how exposure to early life stress may help provide resistance to future stressors.

However, several recent studies exploring the contribution of early adverse experiences to mental health concerns during the pandemic have reported contradictory results. Doom et al. (24) reported that early adverse experiences were directly associated with higher levels of depressive symptoms in students during the COVID-19 pandemic. Kim et al. (18) reported preliminary evidence that adults with more severe childhood traumas may exhibit worse depressive symptoms due to greater COVID-19 risk perception during the lockdown in South Africa, indicating an interaction between childhood trauma and adult stress sensitivity. Guo et al. (27) reported that in a Chinese adolescent population, pre-pandemic maltreatment experiences exaggerated PTSD symptoms in response to COVID-19. In accordance with these studies, this study also found a positive correlation between childhood traumas and the severity of depressive symptoms at both the baseline and after HQ. Meanwhile, this study also revealed positive correlations between childhood traumas and the severity of prodromal psychotic symptoms and overall mental health levels. Notably, all the correlations were generally weaker after HQ than at the baseline (Figure 2A), indicating a buffering effect of HQ on the adverse effects of childhood traumas. Furthermore, we found that the amounts of changes in depressive, prodromal psychotic, and overall symptoms before and after HQ were significantly different between college students with and without childhood traumas. Namely, the students with childhood traumas had a greater decrement in all measured psychiatric symptoms (Figure 1).

Notably, all the participants in this study were home quarantined for 3 months with their core family members before the follow-up survey. The significant changes in living environments and surrounding interpersonal relationships from school to family may account for the buffering effects of HQ on the adverse effects of childhood traumas. Because it has been found that in a school context with similar aged peers from a wide range of backgrounds, the college students with disadvantages in interpersonal comparison may experience more Personal Relative Deprivation (PRD), which was indicated to be associated with childhood maltreatment and poorer mental health (51–53). On the contrary, staying with family members during the pandemic has been proven to protect college students’ mental health (54), indicating that the environment shifts from school to family might decrease the PRD of students with childhood traumas and hence attenuated all measured psychiatric symptoms after the HQ.

Intriguingly, we found that the decrement of depressive symptoms, prodromal psychotic symptoms, and overall psychiatric symptoms were negatively correlated with the baseline social support (Figure 3B). The multilinear regression analysis confirmed these findings when controlling for potential confounding variables (age, sex, place of residence, and percentage of grades). A further constructed SEM indicated that the total effects of childhood trauma on decreased psychiatric symptoms were partly mediated by lower baseline social support (Figure 3). In accordance with previous reports that social supports was negatively associated with PRD (52), and may buffer the devastating effects of PRD on depressive symptoms (55). This could be explained by a relatively smaller decrease in perceived social supports from school or greater increase in perceived supports from the family for the students with childhood traumas in the process of school-to-family environment shifting during HQ. Indeed, HQ may restrict social interactions within only the family members. This restriction may also have brought with it opportunities to rebuild family support systems and enhance family cohesion, which may mitigate the adverse effects of childhood parental neglect and abuse.

In general, childhood traumas, including physical and emotional abuse and neglect and sexual abuse were associated with a higher risk of developing multiple psychiatric symptoms, including increased frequency and severity of psychotic, emotional, obsessive–compulsive symptoms, and interpersonal issues in adulthood (29, 30, 56–58). Personal relative deprivation has been indicated to mediate or aggravate the devastating effects of childhood maltreatment on many kinks of psychiatric disorders and behavioral problems (53, 59, 60). This effects may be more obvious in developing countries, where the socio-economic differentiation and income inequalities were more severe (61). Indeed, we found significant correlations between all CTQ factors and the decrement of depressive and prodromal psychotic symptoms in a Chinese college student population after the HQ. In accordance with a nationally representative survey of 34,653 US adults suggesting EA as the strongest factor associated with both the internalizing and externalizing psychopathology latent variables underlying many mental disorders (62), we also found that EA was most strongly correlated with all of the psychiatric symptoms in this study. Unlike other CTQ factors, EA may be mainly derived from harsh parenting and have long-lasting effects on self-esteem and perceived relative deprivation. As Kessler *et al* concluded that ‘childhood adversities associated with maladaptive family functioning (e.g., child abuse, neglect) were the strongest predictors of mental disorders’, and that ‘there is little specificity across disorders’ after analyzing data from 21 countries, indicating a nonspecific effects of childhood traumas on the risk of mental disorders in adulthood (63).

## Limitations and strengths

This study has several strengths and limitations. To the best of this author’s knowledge, by making use of an ongoing college student mental health survey and explicit baseline psychiatric symptom data, this is the first study showing that the dynamic changes of depressive and prodromal psychotic symptoms before and after HQ were modulated by prior childhood traumas and social support. Importantly, after a relatively long period of family reunion, it was found that there was a general decrement of prodromal psychotic symptoms, which was found to be a predictor of the subsequent full onset of psychotic disorders, which have no suitable treatment medications in a non-clinical setting (64). Furthermore, most of the reported studies on the influence of the COVID-19 pandemic on mental health used convenient web-based samples, which may have brought in a sampling bias that individuals with self-estimated mental health concerns were more prone to respond. Notably, the participants in this study were derived from a school-managed first-year college student mental health survey program, in which the students were required to finish a mental health screening questionnaire, and the participants were recruited through the in-site introduction of this study by a mental health teacher working in the university. As a result, this study sample may have been more representative of the entire college student population and could have provided more unbiased information on their mental health conditions.

Due to ethical reasons, this study could not have a control group that was not home quarantined during the pandemic. Also, the baseline survey was conducted 17 months before the HQ, which may have introduced some confounding factors into the dynamic changes in psychiatric symptoms. For instance, academic performance has been known to impact the mental health status of first-year college students (65). To mitigate the effects of academic performance on the changes in mental health, we included the variable “percentage of grades” in the partial correlation and multilinear regression analyses. It is also worth noting that the baseline mental health survey was conducted during a stressful period, the beginning stage of the first-year students’ university life, which may have exaggerated the prevalence and severity of psychiatric symptoms. However, this exaggeration may have been partly controlled by the follow-up survey, which was conducted when schools reopened, thus mitigating the stress related to the home-to-school transition. Another limitation of this study is that the respondents were exclusively from 10 colleges within a comprehensive university in Yunnan Province. This may have resulted in a selection bias and therefore, may not accurately represent the entire college student population in China.

## Conclusion

This study suggests that the depressive, prodromal psychotic and total psychiatric symptoms of the college students were generally diminished after HQ during the COVID-19 pandemic, and the adverse effects of childhood trauma on mental health status, especially on the prodromal psychotic symptoms were partially blunted during the HQ, which may reduce the subjective and objective personal relative deprivation through full lock-down and overall social isolation. Meanwhile, prior social support may have been unavailable during HQ in the COVID-19 pandemic, thus making the participants previously at a higher order of social ladders feel some degree of loss and loneliness, leading to deteriorating mental health as a result. These results suggest the potential beneficial effects of family context on mental wellbeing and may be informative for making decisions on the location of future quarantines in pandemics.

## Data availability statement

The original contributions presented in the study are included in the article/supplementary material, further inquiries can be directed to the corresponding author.

## Ethics statement

The studies involving human participants were reviewed and approved by Institutional Review Board of the First Affiliated Hospital of Kunming Medical University (2018-L-41). The patients/participants provided their written informed consent to participate in this study.

## Author contributions

WH, QW, CH, and WL: conception and design of the research. WH, QH, YB, and RW: acquisition of data. QW, RW, NZ, CH, and WL: analysis and interpretation of the data. WH and WL: statistical analysis. WL: obtaining financing. WH and QW: writing of the manuscript. YB, CH, and WL: critical revision of the manuscript for intellectual content. All authors contributed to the article and approved the submitted version.

## Funding

This work was supported by grants from the National Natural Science Foundation of China (No. 81860253), Shanghai Pudong New Area Health Committee Disciplinary Leader Training Program (No. PWRd2021-06), Pudong New Area Science and Technology Development Fund (No. PKJ2021-Y17 and PKJ2022-Y77) the Key Clinical Discipline Project of Shanghai Pudong (No. PWZzk2022-19 and PWYgy2018-10).

## Conflict of interest

The authors declare that the research was conducted in the absence of any commercial or financial relationships that could be construed as a potential conflict of interest.

## Publisher’s note

All claims expressed in this article are solely those of the authors and do not necessarily represent those of their affiliated organizations, or those of the publisher, the editors and the reviewers. Any product that may be evaluated in this article, or claim that may be made by its manufacturer, is not guaranteed or endorsed by the publisher.

## Footnotes

^1^ https://www.wjx.cn/

## Abbreviations

COVID-19: Coronavirus disease 2019
HQ: Home quarantine
PHQ-9: Patient health questionnaire-9
PQ-16: 16 item prodromal questionnaire
SCL-90: Symptom checklist-90
CTQ: Childhood trauma questionnaire
SSRS: Social support rating scale
SE: Standard error
PA: Physical abuse
PN: Physical neglect
EA: Emotional abuse
EN: Emotional neglect
SA: Sexual abuse
N/A: Not available
PRD: Personal relative deprivation
